# P061 Reynaud’s syndrome - an unusual manifestation of giant cell arteritis

**DOI:** 10.1093/rap/rkag113.091

**Published:** 2026-11-03

**Authors:** Lennart Marahrens, Jemima Sneddon

**Affiliations:** St George’s University Hospitals NHS Foundation Trust, London, United Kingdom; Homerton Healthcare NHS Foundation Trust, London, United Kingdom

## Abstract

**Introduction:**

Giant cell arteritis (GCA) without cranial involvement is a challenging diagnosis due its non-specific presentation with fever, fatigue, and weight loss in the absence of headache, scalp tenderness, or visual loss. Stenotic disease of the aorta and its major branches, whilst more typically seen in Takayasu’s arteritis, has been described in GCA, and classically results in limb claudication. We report a case of GCA presenting with Reynaud’s phenomena in the hands secondary to bilateral stenoses of the axillary and subclavian arteries. This highlights the diverse nature of the disease and need for a high clinical suspicion in the appropriate context.

**Case description:**

We report on a 66-year-old male. He has a past medical history of atrial fibrillation with previous ablation and hypercholesterolaemia and works as a professional musician. The patient initially presented to his general practitioner with several months of generalised headaches, weight loss, and fatigue. Subsequently, he developed bilateral upper arm claudication on exertion and significant Reynaud’s symptoms with classical symptoms of triphasic colour change and pain in both hands on minimal cold exposure. He underwent basic blood work which was unremarkable expect for a raised CRP of 66mg/L. The patient was referred to the acute medical unit by his GP with suspected infective endocarditis. Pertinent to the presentation with Reynaud’s, there were no other features of an autoimmune connective tissue disease. Examination of the temporal arteries was normal and the patient denied jaw claudication.

The patient underwent CT of his thorax, abdomen, and pelvis which showed generalised mural thickening throughout the aorta and aortic arch vessels, in particular the subclavian arteries bilaterally, with no evidence of malignancy. In light of this, an ultrasound doppler was requested which showed significant inflammation of the subclavian and axillary arteries with >75% flow limiting stenosis of the axillary arteries bilaterally but patent radial and ulnar arteries. PET CT confirmed increased uptake in the thoracoabdominal aorta, subclavian arteries, brachial arteries, iliofemoral arteries, and carotid arteries. Investigations for secondary causes of large vessel vasculitis, including syphilis serology, TB QuantiFERON, and IgG4 levels were unremarkable. ANA, ANCA, RF, anti-CCP, and serum protein electrophoresis were normal.

The patient was commenced on high dose oral prednisolone, methotrexate, and tocilizumab. There was significant clinical improvement in his symptoms with near-normalisation of his inflammatory markers. Currently, the patient is awaiting evaluation of his stenotic disease by vascular surgery and remains on a prednisolone taper and in follow up with rheumatology.

**Discussion:**

This case demonstrates an unusual presentation of GCA, with severe Raynaud’s phenomenon as a presenting feature. Although constitutional symptoms and upper limb claudication are recognised manifestations of GCA, Raynaud’s phenomenon is rarely described and may in the presence of fevers and weight loss lead to a consideration of connective tissue disease (CTD).

The combination of unexplained constitutional symptoms and elevated inflammatory markers prompted cross-sectional imaging, which demonstrated large vessel vasculitis. This again highlights the often non-specific presentation in GCA. This patient’s Reynaud’s is likely secondary to significant stenoses of the axillary arteries as well as aortic inflammation leading to impaired perfusion of the distal circulation, making the hands more susceptible to cold-induced vasospasm. Non-immunosuppressive treatments such as calcium channel blockage or prostaglandins could be considered as adjunct therapies.

This case highlights the importance of considering GCA in patients presenting with atypical vascular symptoms, particularly when accompanied by systemic inflammation. Reynaud’s symptoms in patients with GCA have previously been described in case reports and may represent an under-recognised manifestation. Evaluation for GCA should be considered in patients with new Reynaud’s syndrome in older patients, where new presentations of CTD are less common. Prompt diagnosis is essential to reduce the risk of progressive arterial stenosis and irreversible ischaemic complications.

Treatment with glucocorticoids, methotrexate, and tocilizumab resulted in rapid improvement. However, potentially persistent structural arterial disease necessitates ongoing vascular surveillance and multidisciplinary management. This case broadens the recognised clinical spectrum of GCA and serves as a reminder that severe Raynaud’s phenomenon may, albeit rarely, represent the initial manifestation of large vessel vasculitis rather than CTD or primary Reynaud’s.

**Key learning points:**

• Firstly, GCA should remain an important differential diagnosis in patients presenting with constitutional symptoms, elevated inflammatory markers, and upper limb claudication, even in the absence of cranial features suggestive of giant cell arteritis. Reliance on the classical cranial phenotype alone may delay recognition of large vessel disease.

• Secondly, severe or new-onset Raynaud’s phenomenon in older adults warrants careful evaluation for secondary causes. While connective tissue diseases are commonly considered, this case demonstrates that significant proximal arterial inflammation and stenosis from LVV may present with prominent Raynaud’s symptoms. The coexistence of exertional arm claudication and Raynaud’s phenomenon should prompt consideration of structural arterial pathology in addition to vasospastic disorders.

• Thirdly, a low threshold for further investigation with CT and/or PET scan is important in establishing the diagnosis and defining disease extent in non-cranial GCA. Cross-sectional imaging identified inflammatory changes within the aorta and its major branches, vascular ultrasound demonstrated significant arterial stenoses with flow limitations, and FDG PET-CT confirmed active large vessel inflammation.

• Finally, early recognition and prompt initiation of immunosuppressive therapy can result in rapid clinical and biochemical improvement. However, suppression of vascular inflammation does not necessarily reverse established arterial stenoses, highlighting the need for ongoing assessment of vascular complications and multidisciplinary management involving rheumatology, vascular surgery, and radiology. Careful follow-up is important to distinguish active inflammatory disease from structural vascular damage and to guide decisions regarding further immunosuppression or vascular intervention.

